# Subversion of Immunity by *Leishmania amazonensis* Parasites: Possible Role of Phosphatidylserine as a Main Regulator

**DOI:** 10.1155/2012/981686

**Published:** 2012-02-02

**Authors:** Joao Luiz Mendes Wanderley, Jaqueline França Costa, Valéria Matos Borges, Marcello Barcinski

**Affiliations:** ^1^Universidade Federal do Rio de Janeiro, Pólo Universitário Macaé, Brazil; ^2^Centro de Pesquisa Gonçalo Moniz, Fundação Oswaldo Cruz, Salvador, BA, Brazil; ^3^Faculdade de Medicina, Universidade Federal da Bahia, Salvador, BA, Brazil; ^4^Instituto Nacional de Ciência e Tecnologia de Investigação em Imunologia, Salvador, BA, Brazil; ^5^Laboratório de Biologia Celular, Instituto Oswaldo Cruz, Rio de Janeiro, RJ, Brazil

## Abstract

*Leishmania amazonensis* parasites cause progressive disease in most inbred mouse strains and are associated with the development of diffuse cutaneous leishmaniasis in humans. The poor activation of an effective cellular response is correlated with the ability of these parasites to infect mononuclear phagocytic cells without triggering their activation or actively suppressing innate responses of these cells. Here we discuss the possible role of phosphatidylserine exposure by these parasites as a main regulator of the mechanism underlying subversion of the immune system at different steps during the infection.

## 1. *Leishmania* Parasites

*Leishmania *parasites are heteroxenous kinetoplastid protozoan organisms, which undergo complete differentiation upon a cycle of proliferation/differentiation in the midgut of phlebotomine sand flies followed by the transmission of infective metacyclic promastigotes [1, 2] to mammalian hosts during the insect blood meal. Once infecting mammalian hosts, these organisms, from free-living protozoa, become obligate intracellular parasites, residing and proliferating inside phagolysosomes of mononuclear phagocytic cells as amastigote forms. 

In humans, *Leishmania *parasites can cause a broad spectrum of clinical manifestations from mild, self-resolving skin diseases to potentially fatal, disseminated visceral diseases. The outcome of the infection is dependent on multiple, interdependent factors, such as vector species, parasite species and strain, genetic background, and immunological status of the host. There are two main groups of parasites, stratified upon the clinical outcome of the infection: the ones capable of causing tegumentar and the ones capable of causing visceral diseases. In both cases, disease is initiated by the bite of an infected sand fly, followed by the generation of a skin lesion, mainly caused by the inflammatory response induced on that site. In some cases the disease is confined to the skin or mucosal tissues, and is termed cutaneous (CL) or muco-cutaneous (MCL) leishmaniasis, respectively. In addition, diffuse cutaneous leishmaniasis (DCL) occurs when the parasite disseminates causing the appearance of multiple skin lesions, in distal sites relative to the transmission site [3]. In a similar way, in visceral leishmaniasis, there is parasite dissemination through blood and lymphatic vessels from the initial lesion site. However, these parasites establish in organs that comprise important populations of mononuclear phagocytes, such as bone marrow, spleen, and liver [4]. Among the clinical manifestations observed in humans with the tegumentary disease, diffuse and mucocutaneous leishmaniasis are the most severe forms. In both cases, most patients were found in the South and Central America, associated with *L. amazonensis *infection for DCL and *L. braziliensis *infection for MCL.

### 1.1. Diffuse Cutaneous Leishmaniasis

Diffuse cutaneous leishmaniasis (DCL) is a rare clinical manifestation and is characterized by the appearance of several nonulcerated nodular skin lesions, uncontrolled parasite proliferation, an inefficient cellular immune response against parasite antigens, and resistance to most therapeutic strategies [5, 6]. The lesions are characterized by a dense dermal infiltrate of vacuolated macrophages heavily parasitized. The intense parasitism in the DCL lesions reflects the functional state of macrophages, which are considered permissive. The deficient macrophage activation in DCL hinders the elimination of *Leishmania *resulting in a disorganized inflammatory process, unable to control the infection. The determinants of DCL are multifactorial and may be associated with both immunologic and genetic events of the patient and the pathogenic factors related to the parasite and vector. The participation of factors associated with the parasite has been shown by some authors although it is a point that still remains to be further explored. In this context, the exhibition of markers of apoptosis by the parasite could be a contributing factor during host-parasite interactions as a possible immunosuppressive mechanism of DCL [7].

## 2. Immune Response

### 2.1. Classical *L. major* Infection

Experimental infection models with *Leishmania *parasites have been extensively used as a tool to study immune responses, especially regarding T-cell differentiation [8, 9]. This is due to the fact that inbred mice strains demonstrate specific patterns of susceptibility and resistance to the disease [9, 10] which correlate with the immune response built by these animals. The classical experimental model that generated this knowledge was infection with *L. major *parasites. C57BL/6 mice infected with this parasite develop a Th1 CD4^+^ T-cell response, which is highly effective to activate leishmanicidal and inflammatory mechanisms in macrophages, leading to intracellular parasite destruction. In this case, a skin lesion is formed, which regresses, becoming undetected around 6–8 postinfection [9]. Nevertheless, latent parasites remain in the infected tissue, providing antigens to maintain a protective immune response that prevent reinfections [11]. On the other hand, BALB/c mice infected with the same parasite species and strain developed a Th2 CD4^+^ T-cell response, which is not efficient to promote macrophage classical activation, leading to progressive disease. At the cellular level, this difference is mainly due to the activation of a population of cells that express a highly restricted T-cell receptor, V*β*4 V*α*8, which recognizes the LACK (*Leishmania* homologue of receptors for activated kinase) antigen and rapidly produces IL-4, necessary to deviate the immune response towards Th2 [12]. Currently, it is clear that the proposed model of susceptibility and resistance to *Leishmania* infection is quite reproducible when working with some specific strains of *L. major* though, for other strains and/or species, the picture is relatively more complex. Indeed, effective macrophage activation is the key to control the infection; however, the phenotype displayed by T cells in different situations is not as polarized as observed in the classical model. Actually, there are several papers that suggest that most correlations between CD4^+^ T-cell response and disease development are not straightforward. BALB/c IL-4 receptor knock-out (KO) mice remained susceptible to *L. major *infection when infected with LV39 strain, which seems to be due to an increased production of IL-10 by T cells [13]. C57BL/6 mice infected with a *L. major *strain, isolated from a patient with nonhealing lesions, still developed a Th1 response but displayed a progressive disease [14]. In addition, when infected with the IR173 strain of *L. major*, CD4^+^ T cells from both BALB/c and the resistant mice strain B10.D2 produce IL-4 very rapidly [15]. Other factors such as infection route, number of parasites inoculated, and type of infection (needle versus sand fly inoculation) are crucial to determine the type of response elicited (reviewed in [9]). The complexity of the interactions that determines the clinical and immunological outcome of the disease is much less known, and apparently much more multifactorial in other infection systems, such as the ones that involve *L. amazonensis *infection.

### 2.2. *L. amazonensis* Infection: Beyond the Paradigm

Experimental infection with *L. amazonensis *parasites leads to progressive disease and uncontrolled lesion development in all inbred mouse strains, including those ones that are highly resistant to *L. major *infection. However, there is a gradient of disease severity, ranging from BALB/c mice, which develop a very fast lesion, that ulcerate, generating extensive areas of necrotic tissue, to C3H.HeN mice that still develop nonhealing lesions, however, displaying slow progression rates [16, 17]. Nonetheless, the phenotype displayed by different mouse strains does not correlate with dichotomic Th1/Th2 responses. Actually, in the analyzed mouse strains such as BALB/c, C57BL/6, and C3H.HeN, it was possible to observe CD4^+^ T cells capable of producing different types of cytokines such as Th2 cytokines (IL-4, IL-5, and IL-13), Th1 cytokines (IFN*γ*, and TNF*α*), and regulatory cytokines (TGF*β* and IL-10), which characterizes an unpolarized cellular response [18–20]. Targeted deletion of the *Il4 *or *Il10 *gene [21, 22] causes minimal effects on lesion development and parasite tissue loads as well as treatment of infected mice with IFN*γ* [23] or IL-12 [22]. Interestingly, *L. amazonensis *promastigotes and, especially amastigotes, are able to get through the innate immune response almost unnoticeable. As mentioned before, the main host cell for *Leishmania *proliferation in the mammalian host is the macrophage, which is, together with dendritic cells (DCs), the main antigen presenting cells of the innate immune response. When compared to *L. braziliensis *parasites, for example, *L. amazonensis *parasites are much less capable of triggering the expression of CD40 and CD80 [24], both costimulatory molecules for T-cell activation, and the production of IL-12p40 [24]. Actually, amastigote infection is able to downregulate the expression of MHC class II molecules [25], which, during macrophage infection, is depending on sequestering these molecules inside the parasitophorous vacuole for degradation [26, 27]. During the first week of infection in C57BL/6 mice, chemokines such as CCL5, CCL3, CCL2, CCL4, and CCL11 as well as their receptors, are not upregulated when compared to *L. major *infection, both at the lesion site and draining lymph node [19]. Additionally, amastigote infection downregulates several intracellular pathways that lead to DC activation such as STAT 1, STAT 3 and Erk 1/2 phosphorylation and the expression of the interferon-responsive elements IRF8 and 1, suggesting a global inhibition of inflammatory responses of these cells [25]. The most well-characterized ligand for amastigote recognition and internalization in macrophages is the opsonizing antibodies produced throughout infection. Triggering of Fc receptors on the host cells lead to IL-10 production and has a pathogenic role [28]. These events are necessary to evade the early immune response culminating with the ineffective T-cell response observed in most cases. In parallel to *L. major *infection in BALB/c mice, where the oligoclonal V*β*4 V*α*8 CD4^+^ T-cell population is necessary to the development of susceptibility in the host and is considered as pathogenic T cells, in *L. amazonensis *infection, T cells, in a general way, seem to be highly pathogenic. First of all, there is no clonal or oligoclonal T-cell population involved, since there is no predominance of a single or a group of V*β* chains expressed on T cells that respond to *L. amazonensis *infection [29]. However, the disruption of CD4^+^ T cell effector functions, as observed in recombinase-activating gene KO mice (RAG KO), MHC class II transactivator KO mice (CIITA KO) and nude mice leads to transient resistance to *L. amazonensis *infection, measured by lesion development [30]. In addition, the adoptive transfer of regulatory T cells also restrains pathogenic effector T cells, diminishing lesion size and parasite tissue loads [31]. The mechanisms underlying pathogenic role of T cells for the disease need to be determined.

## 3. Phosphatidylserine Exposure

### 3.1. Homeostasis and Efferocytosis

Phosphatidylserine (PS) is a structural phospholipid present in virtually all membranes and cell types. In normal cells these molecules face the cytoplasmic leaflet of the plasma membrane, whereas during apoptotic cell death these molecules translocate to the outer surface. Once outside the cell, PS becomes one of the ligands recognized by surrounding phagocytes to clear dying cells [32]. However, PS in this model is not just one eat-me signal [33]. PS is the most characterized tickling [34] ligand of apoptotic cells, which means that PS provides the signals for the phagocyte to activate immunosuppressive and anti-inflammatory mechanisms. PS recognition is mandatory to prevent the establishment of a response to the self-antigens engulfed by these cells during apoptotic cell clearance and to avoid triggering inflammatory responses, especially during the embryogenesis, when massive amounts of apoptotic cells are generated and therefore, cleared [35–37], but also in adults to prevent inflammatory immunopathologies [32]. The intracellular events, receptors, and soluble factors involved in this mechanism are still being deciphered and are not the focus of this discussion. However, the effects of PS recognition in macrophages and DCs have a direct impact in immune responses. Apoptotic cells actively induce the production of the anti-inflammatory cytokines TGF*β*, PGE2, and PAF [35] and actively inhibit the production of TNF*α* and IL-1*β*, even upon LPS challenge [35, 38]. Recognition of apoptotic cells also decreases the expression of several activation markers and costimulatory molecules by both human and murine DCs [39, 40] and regulates the expression of cytokines involved with T-cell differentiation at the transcriptional level [41, 42]. At the single cell level, DCs that ingested an apoptotic cell and bacteria at the same time are able to discern between them and only present bacterial antigens. This is possible because the generation of peptide-MHC class II complexes is controlled by toll-like receptors (TLRs) in a strictly phagosome autonomous manner. Since apoptotic cells do not trigger TLR activation, the generation of stable complexes is inhibited or abrogated [43]. All these effects are fundamental to maintain homeostasis and comprehend the last step of the efferocytosis [44] or apoptotic cell clearance. However, it seems that intracellular parasites elegantly make use of these mechanisms to establish in the host [45–47]. Furthermore, some parasites mimic the features of apoptotic cells to avoid host immune response, as discussed in the next section.

### 3.2. Conserved Immune-Evasion Mechanism?

One of the most common PCD phenotypes is phosphatidylserine (PS) exposure, which can be observed upon chemotherapy, starvation, and heat shock conditions in several unicellular organisms [48–51] or is actively displayed in normal conditions [52]. Our group observed that lesion-derived amastigotes of *L. amazonensis *actively expose high levels of PS, and by blocking this molecule there is a drastic decrease in the ability of these parasites to infect and establish in the macrophages [52]. These parasites are viable and capable of differentiating into promastigote forms *in vitro *(unpublished data) and inside the sand fly vector [53] and to infect macrophages and mice [52, 54] and did not display other markers of PCD. Therefore we denominated this mechanism as apoptotic mimicry. PS exposure on amastigotes of *L. amazonensis *occurs in virtually 100% of the parasites; however, the amount of PS molecules depends on the infected host. Parasites obtained from BALB/c mice expose higher amounts of PS than the ones obtained from C57BL/6 mice [54]. This observation demonstrates that the amount of PS at the surface of the amastigotes has a positive correlation with the severity of the disease and suggests that the host is able to modulate this phenotype of the parasite. Following our description several other groups demonstrated the role of PS exposure and recognition in different infection models. Blood and cell-derived trypomastigotes of *Trypanosoma cruzi *are able to expose PS, in contrast with epimastigotes, which are not. In addition, infection with PS-exposing trypomastigote forms induces Smad nuclear translocation and inducible nitric oxide synthase inhibition (iNOS), suggesting an autocrine modulation of the host cell dependent on TGF*β* [55]. It is interesting to note that, among all *T. cruzi *parasite stages, only the ones that are infective for mammalian cells evolve the ability to expose PS, suggesting the presence of an evolutionary link between PS exposure and the ability to infect host cells. Similarly, *Toxoplasma gondii *peritoneal tachyzoites expose PS at their surface, and the recognition of this molecule seems to be necessary to downmodulate iNOS expression and activity upon macrophage infection [56]. More recently, several papers have demonstrated the role of exposed PS molecules for the infection by enveloped viral particles. For human immunodeficiency Virus-1 (HIV-1), PS at the viral envelope is a cofactor for monocyte infection [57]; in vaccinia virus infection, PS recognition modulates the activity of proteins involved in cytoskeleton reorganization such as p21-activated kinase (PAK) and the small Rho GTPase Rac, leading to increased macropinocytic activity and uptake of viral particles [58]. In addition, PS exposure by tumor cell, microvesicles shed by transformed cells, or endothelial cells in the intratumor environment seems to be involved in different events in tumor development, maintenance, and metastasis [59, 60]. This knowledge stimulated some researchers to evaluate the efficacy of anti-PS antibodies to treat viral and tumoral diseases. Actually the results so far are promising. In murine models of Lassa fever (Pichinde virus) or murine cytomegaloviruses the treatment efficacy was very high, reaching complete cure (total absence of detectable viral loads) in combination with available antiaviral drugs [61]. For experimental tumoral disease, lung cancer, pancreatic tumors, and glioblastomas were efficiently treated, decreasing tumor growth and metastasis in some cases or potentiating the effect of chemo- and radio-therapies [62–64].

### 3.3. *Leishmania amazonensis* Infection: New Insights

Our group has been committed to study the role of PS exposure on the surface of different isolates of *L. amazonensis*. We worked with the hypothesis that *L. amazonensis *isolates from DCL patients would have higher PS exposure compared with localized cutaneous leishmaniasis (LCL), and this would contribute to macrophage deactivation, favoring parasite replication. For this, we compared PS exposure in *L. amazonensis *isolates from DCL clinical cases in the active phase of the disease, reported in Maranhão state in Brazil, to those isolated from LCL patients of clinical cases from Bahia. The results indicate that the isolates obtained from DCL patients indeed displayed more PS than isolates from LCL patients at early times postinfection. In addition, isolates from DCL patients were more infective than the ones obtained from LCL patients (França-Costa et al., unpublished results). On the other hand, independent of parasite strain analyzed, the parameters of infectivity correlated positively with the exposure of PS in the parasites. These data suggest that in human infections the pattern observed in mice when comparing BALB/c versus C57BL/6 mice is maintained. However, it is necessary to investigate the mechanisms by which the recognition of PS on the surface of the isolates of *L. amazonensis *deactivate the macrophage response. Particularly, it would be necessary to evaluate whether freshly isolated parasites display this phenotype to validate our analysis made on amastigotes derived from macrophages infected *in vitro *with cultured promastigote parasites isolated from human lesions. We believe that understanding the dynamics of PS expression, along with identification of the mechanisms involved in the immunosuppression of DCL patients, can result in therapeutic targets for intervention in the immunopathogenesis of this chronic and severe form of leishmaniasis. 

**Figure 1:**
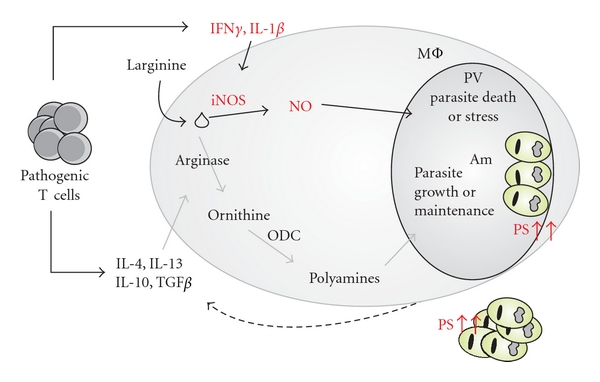
PS exposure on intracellular amastigotes of *L. amazonensis: *hypothesis for T-cell-dependent modulation. T cells primed by leishmanial antigens display a pathogenic phenotype, characterized by the production of unpolarized cytokines [18, 31]. These cytokines are able to activate both iNOS- and arginase 1-dependent intracellular macrophage pathways (Wanderley, JL et al. unpublished). In this environment, amastigotes receive stress from iNOS-derived nitric oxide (NO) which triggers high levels of surface PS on the parasite (Wanderley, JL et al. unpublished). Simultaneously, arginase 1 is also induced, and the outcome of this activation is an increase in polyamine intracellular levels [65]. Polyamines are indispensable for parasite survival and proliferation, maintaining them even in the presence of NO (Wanderley, JL et al. unpublished, [63]). Upon macrophage disruption, highly infective PSHIGH amastigotes are released, being capable of infecting new host cells and of spreading the anti-inflammatory signals derived from PS recognition (dashed arrow). PV: parasitophorous vacuole, ODC: ornithine decarboxylase, iNOS: inducible nitric oxide synthase, MΦ: macrophage, Am: amastigote.

In a similar way we are interested in the immunomodulatory mechanisms underlying PS exposure in different inbred mice strains. For that we are currently evaluating these mechanisms during BALB/c infection, which induces high levels of PS exposure on intracellular amastigotes. We observed that PS exposure is intrinsic to the intracellular parasite and cannot be observed in axenically cultured amastigotes but upregulates very fast after internalization. However, these levels are dramatically increased when infected macrophages are in the presence of previously primed T cells or their soluble products. We confirmed these results by infecting BALB/c nude mice where we observed that the amastigotes obtained from these mice display minimal levels of PS, which are completely restored if we adoptively transfer primed CD4^+^ T cells to nude mice (Wanderley et al. unpublished results). Interestingly, these data indicate that one possible role for the previously reported pathogenic T cells [31] would be to induce PS exposure on intracellular amastigotes and, therefore, contributing to the generation of highly infective parasites. The T-cell-dependent PS exposure on amastigotes seems to be dependent on the induction of iNOS expression on host macrophages, and parasite survival is dependent on the concomitant induction of arginase 1 expression (Wanderley et al. unpublished results). We propose that high levels of PS exposure are induced by parasite stress delivered by iNOS activity. In this case, it is still unknown whether PS exposure on amastigotes is indeed a phenotype triggered by PCD or a specific process involving modulation of PS translocation. Under PS-inducting conditions, macrophages express high levels of arginase 1 (Wanderley et al. unpublished results), that is the enzyme necessary to produce ornithine, the precursor of polyamines. In this situation, polyamines could protect the parasite from the iNOS-dependent stress, stimulating parasite growth [66, 67] and increasing DNA stabilization [68, 69]. We understand that the unique characteristics of the T-cell response to *L. amazonensis *infection contribute to the generation of a perfect environment to stimulate and maintain increased levels of PS on the surface of intracellular parasites. Probably the balance observed in infected BALB/c mice, when disrupted, leads to the differences observed among different mouse strains. In Figure 1 we summarized our hypothesis regarding the T-cell-dependent modulation of PS exposure on intracellular amastigotes of *L. amazonensis*.

## 4. Final Remarks

The observation of PS exposure as a strategy to evade the immune system and persist in the mammalian host, made initially in the experimental model of *L. amazonensis *infection, was a breakthrough since it stimulated different groups around the world to look for the possibilities for basic and applied research on the field. Our group is still studying the immunological, cellular, and molecular mechanisms underlying control of PS exposure in parasites and the effects of its recognition by parasitized cells and organisms. We believe that this could be a major strategy in different systems where avoidance from immune surveillance is necessary to establish a disease.

## References

[1] Sacks DL, Perkins PV (1984). Identification of an infective stage of *Leishmania* promastigotes. *Science*.

[2] Sacks DL, Perkins PV (1985). Development of infective stage *Leishmania* promastigotes within phlebotomine sand flies. *American Journal of Tropical Medicine and Hygiene*.

[3] Silveira FT, Lainson R, Corbett CEP (2005). Further observations on clinical, histopathological, and immunological features of borderline disseminated cutaneous leishmaniasis caused by *Leishmania (Leishmania)* amazonensis. *Memorias do Instituto Oswaldo Cruz*.

[4] Guerin PJ, Olliaro P, Sundar S (2002). Visceral leishmaniasis: current status of control, diagnosis, and treatment, and a proposed research and development agenda. *Lancet Infectious Diseases*.

[5] Perrella Balestieri FM, Pires Queiroz AR, Scavone C, Assis Costa VM, Barral-Netto M, Abrahamsohn IDA (2002). *Leishmania (L.) amazonensis*-induced inhibition of nitric oxide synthesis in host macrophages. *Microbes and Infection*.

[6] Convit J, Ulrich M, Fernandez CT (1993). The clinical and immunological spectrum of American cutaneous leishmaniasis. *Transactions of the Royal Society of Tropical Medicine and Hygiene*.

[7] Barral A, Costa JML, Bittencourt AL, Barral-Netto M, Carvalho EM (1995). Polar and subpolar diffuse cutaneous leishmaniasis in Brazil: clinical and immunopathologic aspects. *International Journal of Dermatology*.

[8] Mougneau E, Bihl F, Glaichenhaus N (2011). Cell biology and immunology of *Leishmania*. *Immunological Reviews*.

[9] Sacks D, Noben-Trauth N (2002). The immunology of susceptibility and resistance to *Leishmania major* in mice. *Nature Reviews Immunology*.

[10] Kellina OI (1973). Differences in the sensitivity of inbred mice of different lines to *Leishmania tropica* major. *Meditsinskaia Parazitologiia i Parazitarnye Bolezni*.

[11] Belkaid Y, Hoffmann KF, Mendez S (2001). The role of interleukin (IL)-10 in the persistence of *Leishmania major* in the skin after healing and the therapeutic potential of anti-IL-10 receptor antibody for sterile cure. *Journal of Experimental Medicine*.

[12] Launois P, Maillard I, Pingel S (1997). IL-4 Rapidly produced by V*β*4 V*α*8 CD4^+^ T cells instructs Th2 development and susceptibility to *Leishmania major* in BALB/c mice. *Immunity*.

[13] Noben-Trauth N, Lira R, Nagase H, Paul WE, Sacks DL (2003). The relative contribution of IL-4 receptor signaling and IL-10 to susceptibility to *Leishmania major*. *Journal of Immunology*.

[14] Anderson CF, Mendez S, Sacks DL (2005). Nonhealing infection despite Th1 polarization produced by a strain of *Leishmania major* in C57BL/6 mice. *Journal of Immunology*.

[15] Stetson DB, Mohrs M, Mallet-Designe V, Teyton L, Locksley RM (2002). Rapid expansion and IL-4 expression by *Leishmania*-specific naive helper T cells in vivo. *Immunity*.

[16] Silva-Almeida M, Carvalho LOP, Abreu-Silva AL, d’Escoffier LN, Calabrese KS (2010). *Leishsmania (Leishmania) amazonensis* infection: muscular involvement in BALB/c and C3H.HeN mice. *Experimental Parasitology*.

[17] Watanabe Y, Hamaguchi-Tsuru E, Morimoto N (2004). IL-5-induced eosinophils suppress the growth of *Leishmania amazonensis* in vivo and kill promastigotes in vitro in response to either IL-4 or IFN-*γ*. *DNA and Cell Biology*.

[18] Ji J, Sun J, Qi H, Soong L (2002). Analysis of T helper cell responses during infection with *Leishmania amazonensis*. *American Journal of Tropical Medicine and Hygiene*.

[19] Ji J, Sun J, Soong L (2003). Impaired expression of inflammatory cytokines and chemokines at early stages of infection with *Leishmania amazonensis*. *Infection and Immunity*.

[20] Afonso LCC, Scott P (1993). Immune responses associated with susceptibility of C57BL/10 mice to *Leishmania amazonensis*. *Infection and Immunity*.

[21] Jones DE, Ackermann MR, Wille U, Hunter CA, Scott P (2002). Early enhanced Th1 response after *Leishmania amazonensis* infection of C57BL/6 interleukin-10-deficient mice does not lead to resolution of infection. *Infection and Immunity*.

[22] Jones DE, Buxbaum LU, Scott P (2000). IL-4-independent inhibition of IL-12 responsiveness during *Leishmania amazonensis* infection. *Journal of Immunology*.

[23] Barral-Netto M, Von Sohsten RL, Teixeira M (1996). In vivo protective effect of the lectin from Canavalia brasiliensis on BALB/c mice infected by *Leishmania amazonensis*. *Acta Tropica*.

[24] Vargas-Inchaustegui DA, Tai W, Xin L, Hogg AE, Corry DB, Soong L (2009). Distinct roles for MyD88 and toll-like receptor 2 during Leishmania braziliensis infection in mice. *Infection and Immunity*.

[25] Xin L, Li K, Soong L (2008). Down-regulation of dendritic cell signaling pathways by *Leishmania amazonensis* amastigotes. *Molecular Immunology*.

[26] Antoine JC, Jouanne C, Lang T, Prina E, De Chastellier C, Frehel C (1991). Localization of major histocompatibility complex class II molecules in phagolysosomes of murine macrophages infected with *Leishmania amazonensis*. *Infection and Immunity*.

[27] Antoine JC, Lang T, Prina E, Courret N, Hellio R (1999). H-2M molecules, like MHC class II molecules, are targeted to parasitophorous vacuoles of *Leishmania*-infected macrophages and internalized by amastigotes of *L. amazonensis* and *L. mexicana*. *Journal of Cell Science*.

[28] Wanasen N, Xin L, Soong L (2008). Pathogenic role of B cells and antibodies in murine *Leishmania amazonensis* infection. *International Journal for Parasitology*.

[29] Xin L, Wanderley JL, Wang Y, Vargas-Inchaustegui DA, Soong L (2011). The magnitude of CD4^+^ T-cell activation rather than TCR diversity determines the outcome of *Leishmania* infection in mice. *Parasite Immunology*.

[30] Soong L, Chang CH, Sun J (1997). Role of CD4+ T cells in pathogenesis associated with *Leishmania amazonensis* infection. *Journal of Immunology*.

[31] Ji J, Masterson J, Sun J, Soong L (2005). CD4+CD25+ regulatory T cells restrain pathogenic responses during *Leishmania amazonensis* infection. *Journal of Immunology*.

[32] Savill J, Fadok V (2000). Corpse clearance defines the meaning of cell death. *Nature*.

[33] Savill J, Gregory C (2007). Apoptotic PS to phagocyte TIM-4: eat me. *Immunity*.

[34] Hoffmann PR, DeCathelineau AM, Ogden CA (2001). Phosphatidylserine (PS) induces PS receptor-mediated macropinocytosis and promotes clearance of apoptotic cells. *Journal of Cell Biology*.

[35] Fadok VA, Bratton DL, Konowal A, Freed PW, Westcott JY, Henson PM (1998). Macrophages that have ingested apoptotic cells in vitro inhibit proinflammatory cytokine production through autocrine/paracrine mechanisms involving TGF-*β*, PGE2, and PAF. *Journal of Clinical Investigation*.

[36] Fadok VA, McDonald PP, Bratton DL, Henson PM (1998). Regulation of macrophage cytokine production by phagocytosis of apoptotic and post-apoptotic cells. *Biochemical Society Transactions*.

[37] Fadok VA, Voelker DR, Campbell PA, Cohen JJ, Bratton DL, Henson PM (1992). Exposure of phosphatidylserine on the surface of apoptotic lymphocytes triggers specific recognition and removal by macrophages. *Journal of Immunology*.

[38] Voll RE, Herrmann M, Roth EA, Stach C, Kalden JR, Girkontaite I (1997). Immunosuppressive effects of apoptotic cells [9]. *Nature*.

[39] Chen X, Doffek K, Sugg SL, Shilyansky J (2004). Phosphatidylserine regulates the maturation of human dendritic cells. *Journal of Immunology*.

[40] Stuart LM, Lucas M, Simpson C, Lamb J, Savill J, Lacy-Hulbert A (2002). Inhibitory effects of apoptotic cell ingestion upon endotoxin-driven myeloid dendritic cell maturation. *Journal of Immunology*.

[41] Henson PM (2004). Fingering IL-12 with Apoptotic Cells. *Immunity*.

[42] Kim S, Elkon KB, Ma X (2004). Transcriptional suppression of interleukin-12 gene expression following phagocytosis of apoptotic cells. *Immunity*.

[43] Magarian Blander J, Medzhitov R (2006). Toll-dependent selection of microbial antigens for presentation by dendritic cells. *Nature*.

[44] Vandivier RW, Henson PM, Douglas IS (2006). Burying the dead: the impact of failed apoptotic cell removal (efferocytosis) on chronic inflammatory lung disease. *Chest*.

[45] Osorio Y Fortéa J, Prina E, De La Llave E, Lecoeur H, Lang T, Milon G (2007). Unveiling pathways used by *Leishmania amazonensis* amastigotes to subvert macrophage function. *Immunological Reviews*.

[46] Laskay T, van Zandbergen G, Solbach W (2008). Neutrophil granulocytes as host cells and transport vehicles for intracellular pathogens: apoptosis as infection-promoting factor. *Immunobiology*.

[47] Freire-De-Lima CG, Nascimento DO, Soares MBP (2000). Uptake of apoptotic cells drives the growth of a pathogenic trypanosome in macrophages. *Nature*.

[48] Moreira MEC, Del Portillo HA, Milder RV, Balanco JMF, Barcinski MA (1996). Heat shock induction of apoptosis in promastigotes of the unicellular organism *Leishmania (Leishmania) amazonensis*. *Journal of Cellular Physiology*.

[49] Welburn SC, Maudlin I (1997). Control of Trypanosoma brucei brucei infections in tsetse, Glossina morsitans. *Medical and Veterinary Entomology*.

[50] Chose O, Noel C, Gerbod D, Brenner C, Viscogliosi E, Roseto A (2002). A form of cell death with some features resembling apoptosis in the amitochondrial unicellular organism Trichomonas vaginalis. *Experimental Cell Research*.

[51] Arnoult D, Akarid K, Grodet A, Petit PX, Estaquier J, Ameisen JC (2002). On the evolution of programmed cell death: apoptosis of the unicellular eukaryote *Leishmania major* involves cysteine proteinase activation and mitochondrion permeabilization. *Cell Death and Differentiation*.

[52] De Freitas Balanco JM, Costa Moreira ME, Bonomo A (2001). Apoptotic mimicry by an obligate intracellular parasite downregulates macrophage microbicidal activity. *Current Biology*.

[53] Wanderley JLM, da Silva LHP, Deolindo P (2009). Cooperation between apoptotic and viable metacyclics enhances the pathogenesis of leishmaniasis. *PLoS ONE*.

[54] Wanderley JLM, Moreira MEC, Benjamin A, Bonomo AC, Barcinski MA (2006). Mimicry of apoptotic cells by exposing phosphatidylserine participates in the establishment of amastigotes of *Leishmania (L) amazonensis* in mammalian hosts. *Journal of Immunology*.

[55] DaMatta RA, Seabra SH, Deolindo P (2007). *Trypanosoma cruzi* exposes phosphatidylserine as an evasion mechanism. *FEMS Microbiology Letters*.

[56] Seabra SH, De Souza W, Damatta RA (2004). *Toxoplasma gondii* exposes phosphatidylserine inducing a TGF-*β*1 autocrine effect orchestrating macrophage evasion. *Biochemical and Biophysical Research Communications*.

[57] Callahan MK, Popernack PM, Tsutsui S, Truong L, Schlegel RA, Henderson AJ (2003). Phosphatidylserine on HIV envelope is a cofactor for infection of monocytic cells. *Journal of Immunology*.

[58] Mercer J, Helenius A (2008). Vaccinia virus uses macropinocytosis and apoptotic mimicry to enter host cells. *Science*.

[59] Ran S, Downes A, Thorpe PE (2002). Increased exposure of anionic phospholipids on the surface of tumor blood vessels. *Cancer Research*.

[60] Lima LG, Chammas R, Monteiro RQ, Moreira MEC, Barcinski MA (2009). Tumor-derived microvesicles modulate the establishment of metastatic melanoma in a phosphatidylserine-dependent manner. *Cancer Letters*.

[61] Soares MM, King SW, Thorpe PE (2008). Targeting inside-out phosphatidylserine as a therapeutic strategy for viral diseases. *Nature Medicine*.

[62] Beck AW, Luster TA, Miller AF (2006). Combination of a monoclonal anti-phosphatidylserine antibody with gemcitabine strongly inhibits the growth and metastasis of orthotopic pancreatic tumors in mice. *International Journal of Cancer*.

[63] He J, Luster TA, Thorpe PE (2007). Radiation-enhanced vascular targeting of human lung cancers in mice with a monoclonal antibody that binds anionic phospholipids. *Clinical Cancer Research*.

[64] He J, Yin Y, Luster TA, Watkins L, Thorpe PE (2009). Antiphosphatidylserine antibody combined with irradiation damages tumor blood vessels and induces tumor immunity in a rat model of glioblastoma. *Clinical Cancer Research*.

[65] Iniesta V, Carcelén J, Molano I (2005). Arginase I induction during *Leishmania major* infection mediates the development of disease. *Infection and Immunity*.

[66] Iniesta V, Gomez-Nieto LC, Corraliza I (2001). The inhibition of arginase by N*ω*-hydroxy-L-arginine controls the growth of *Leishmania* inside macrophages. *Journal of Experimental Medicine*.

[67] Iniesta V, Gomez-Nieto LC, Molano I (2002). Arginase I induction in macrophages, triggered by Th2-type cytokines, supports the growth of intracellular Leishmania parasites. *Parasite Immunology*.

[68] Rowlatt C, Smith GJ (1981). Ultrastructural studies on chromatin digestion by micrococcal nuclease in the presence of polyamines. *Journal of Cell Science*.

[69] Ha HC, Yager JD, Woster PA, Casero RA (1998). Structural specificity of polyamines and polyamine analogues in the protection of DNA from strand breaks induced by reactive oxygen species. *Biochemical and Biophysical Research Communications*.

